# Employing radiography (X‐rays) to localize lesions in human skeletal remains from past populations to allow accurate biopsy, using examples of cancer metastases

**DOI:** 10.1002/oa.3087

**Published:** 2022-01-11

**Authors:** Piers D. Mitchell, Jenna M. Dittmar

**Affiliations:** ^1^ McDonald Institute for Archaeological Research University of Cambridge Cambridge UK; ^2^ Department of Archaeology University of Aberdeen Aberdeen UK

**Keywords:** aDNA, biopsy, gene mutation, imaging, malignancy, osteoarcheology, proteomics, radiographs

## Abstract

Clinical research into biomolecules from infectious diseases and cancers has advanced rapidly in recent years, with two key areas being DNA analysis and proteomics. If we wish to understand important diseases and their associated biomolecules in past populations, techniques are required that will allow accurate biopsy of lesions in excavated human skeletal remains. While locating lesions visible on the surface of a bone is simple, many lesions such as cancer metastases are located in the medulla of bones, unseen on visual inspection. Here, we use two novel image guided techniques to investigate how plain radiographs may improve accuracy in the localization of lesions within bones from medieval individuals. While both techniques were effective, we found the grid technique required fewer radiographs than the pointer technique to employ and so was responsible for a lower overall radiation dose. We then discuss methods available for biopsy in archeological bone and how the optimal location for the biopsy of malignant lesions will vary depending upon whether the tumor is blastic or lytic in nature. Limitations of this X‐ray guided approach include that not all cancer metastases are visible on plain radiographs, as erosion of cortical bone is frequently required for visualization of lytic metastases using this imaging modality.

## 1 INTRODUCTION

In recent years it has become commonplace for those working in different fields within the archeological sciences to biopsy human skeletal remains. Key specialties that rely on biopsies of bones and teeth include stable isotopes, histology, ancient DNA, and proteomics (Advisory Panel on the Archaeology of Burials in England, 2013). If material representative of host biomolecules is required, then samples are typically acquired from the same location in each individual tested to optimize reproducibility. However, if we need to sample tissue affected by an infectious disease or cancer, then material is needed from wherever in the body that disease process was located. These are known as targeted biopsies.

Cancers are malignant tumors resulting from the uncontrolled division of cells in a tissue. In order to investigate why and how tissues become malignant, there has been a large amount of clinical research on cancer in modern patients that focuses on biomolecules. DNA of the host cancer predisposition genes has shown that some genes increase the risk of developing a number of different cancer types, while others seem mainly associated with cancer in a particular tissue type or organ (Rahman, 2014; Whitworth et al., 2018). Proteomics allows the detection of proteins that are produced by cancers of particular organs and so helps in the diagnosis (Broodman et al., 2017; Ferro et al., 2016; Szász et al., 2017). In a modern clinical setting, biopsy of the cancer is routinely performed in order to enable further investigation of its nature (Criscitiello et al., 2014; Ziv et al., 2016).

Advances in the fields of aDNA and paleoproteomics have opened up the potential for paleopathologists to now apply these techniques to the identification of ancient cancers (Nerlich, 2018). If we are able to determine the past human genome, we can identify gene mutations that would have predisposed an individual to cancer (Feldman et al., 2016). If we take biopsies of ancient cancer lesions, we can use this material to attempt to identify the genetic mutation in the cancer that triggered its formation (Ottini et al., 2011). This can also help us to identify its tissue of origin. Similarly, proteomics of such biopsies has the potential for us to identify proteins from the organ of origin (Bona et al., 2014; Schultz et al., 2007; Shaw et al., 2019). However, such analysis can only be performed if we are able to take accurate biopsies of the correct tissue that contained the cancer during life.

The aim of this study was to develop effective novel image guided techniques to localize pathological lesions present within bones such as the pelvis, femora and spine, so allowing more accurate biopsy of archeological bone. While we use cancer metastases as our examples, the same approach could be used to perform targeted biopsies of lesions caused by a range of different pathologies.

## 2 MATERIALS AND METHODS

**FIGURE 1:**
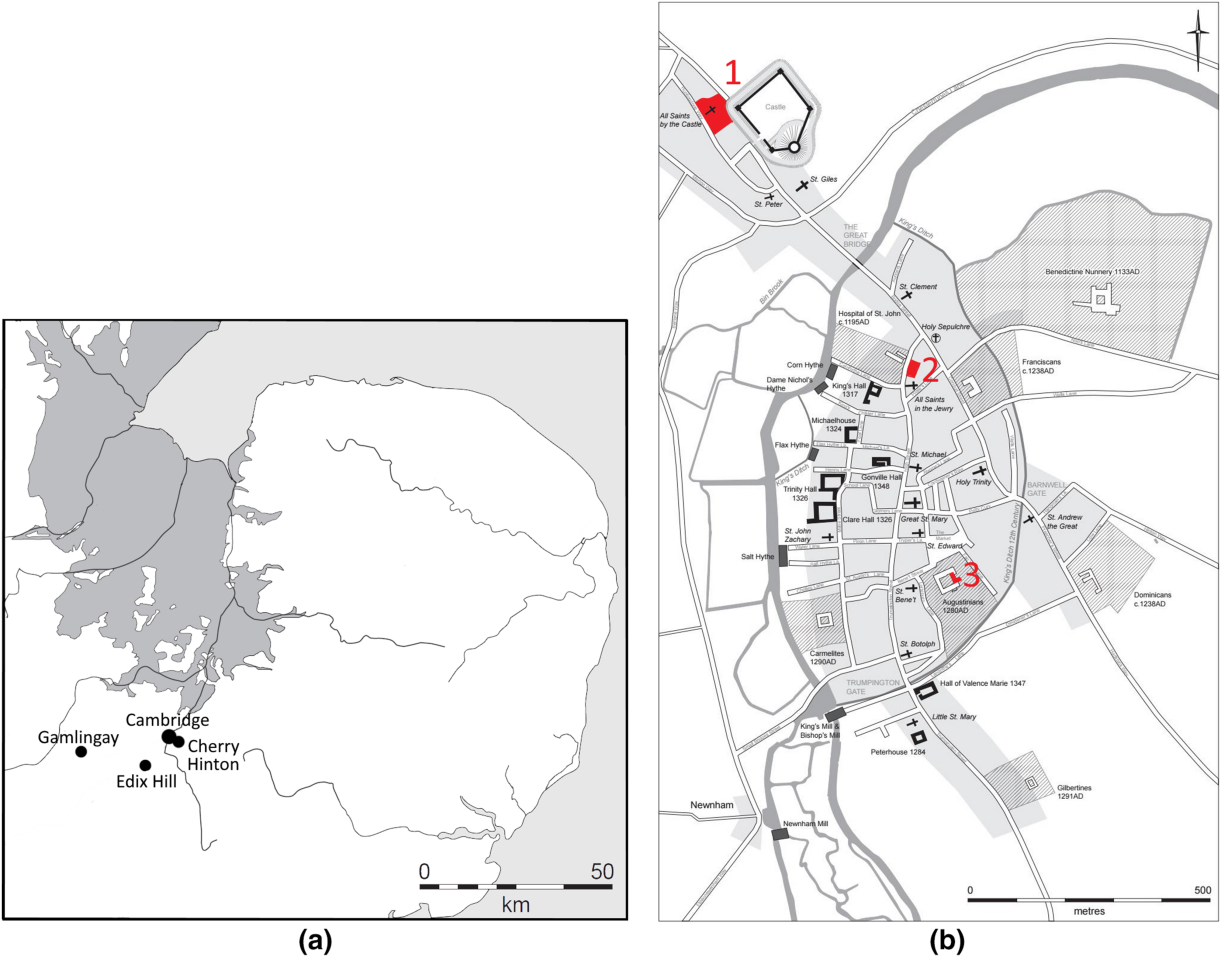
(a) Map showing location of sites in the East of England where the human skeletal remains analyzed for this study were excavated. (b) Plan of sites in Cambridge, where 1 indicates All Saints by the Castle and 2 indicates the Hospital of St. John detached cemetery. Base map produced by Vicki Herring for the After the Plague project [Colour figure can be viewed at wileyonlinelibrary.com]

The bones of five medieval individuals from Cambridge and the surrounding area (United Kingdom) with known cancer metastases were used for this study. These cases have been published in detail elsewhere (Mitchell et al., 2021; Mitchell et al., 2022) and came from the medieval cemeteries at Edix Hill (PSN599), All Saints by the Castle (PSN737 and PSN796), Station Road Gamlingay (PSN807), and the Hospital of St. John the Evangelist (PSN160) (Figure 1). Lesions that had eroded through the cortical surface, and so were visible on inspection, were not included in this study. Those bones with apparently normal external appearance, but with intramedullary lesions visible on plain radiographs, were included in the study.

Imaging was performed by a qualified radiographer, working for the company Reveal Imaging Ltd. Radiation risk assessment was completed prior to the analysis, in order to minimize hazard. Adjustments made included an assessment of the architectural plans for the room where the imaging was to take place, the use of warning signs, ensuring people were at a safe distance from the source of radiation, the use of lead rubber aprons, and positioning the beam towards a solid concrete floor. The as low as reasonably practicable (ALARP) principal, limiting radiation use, was followed. Plain radiographs were taken using a portable DR‐go direct digital X‐ray system. The Xograph DR‐go system uses a Canon Lumix CDXI 35 × 43 cm direct digital radiography plate. The X‐ray tube used was a Sedecal SP4 4‐kW stationary anode high‐frequency generator X‐ray machine. Exposures given followed pre‐existing guidelines established by Reveal Imaging for archeological radiography. The factors were selected relevant to the bone under examination and were normally within the range of 50–55 kV and 1.2–2.5 mAs at a distance of 1 m. DICOM image files were viewed and exported using Merge Efilm (v.3.4.0) software and OsirixX Lite v.12.0.4.

A plain radiograph of the relevant bone containing the metastases was taken (Mitchell et al., 2021; Mitchell et al., 2022). Lesions large enough to be suitable for biopsy were identified and their approximate location noted. In the pelvis, only one view was required as the bone is relatively thin. However, in thicker bones, such as the femur, a lateral view was taken to identify if the metastasis was located anteriorly, posteriorly, or centrally. Biopsy could then be planned from the side where the metastasis was located closest to the bone surface. Two techniques were developed to help accurately localize metastases present within the medulla of the bones:

### 2.1 Grid technique

**FIGURE 2:**
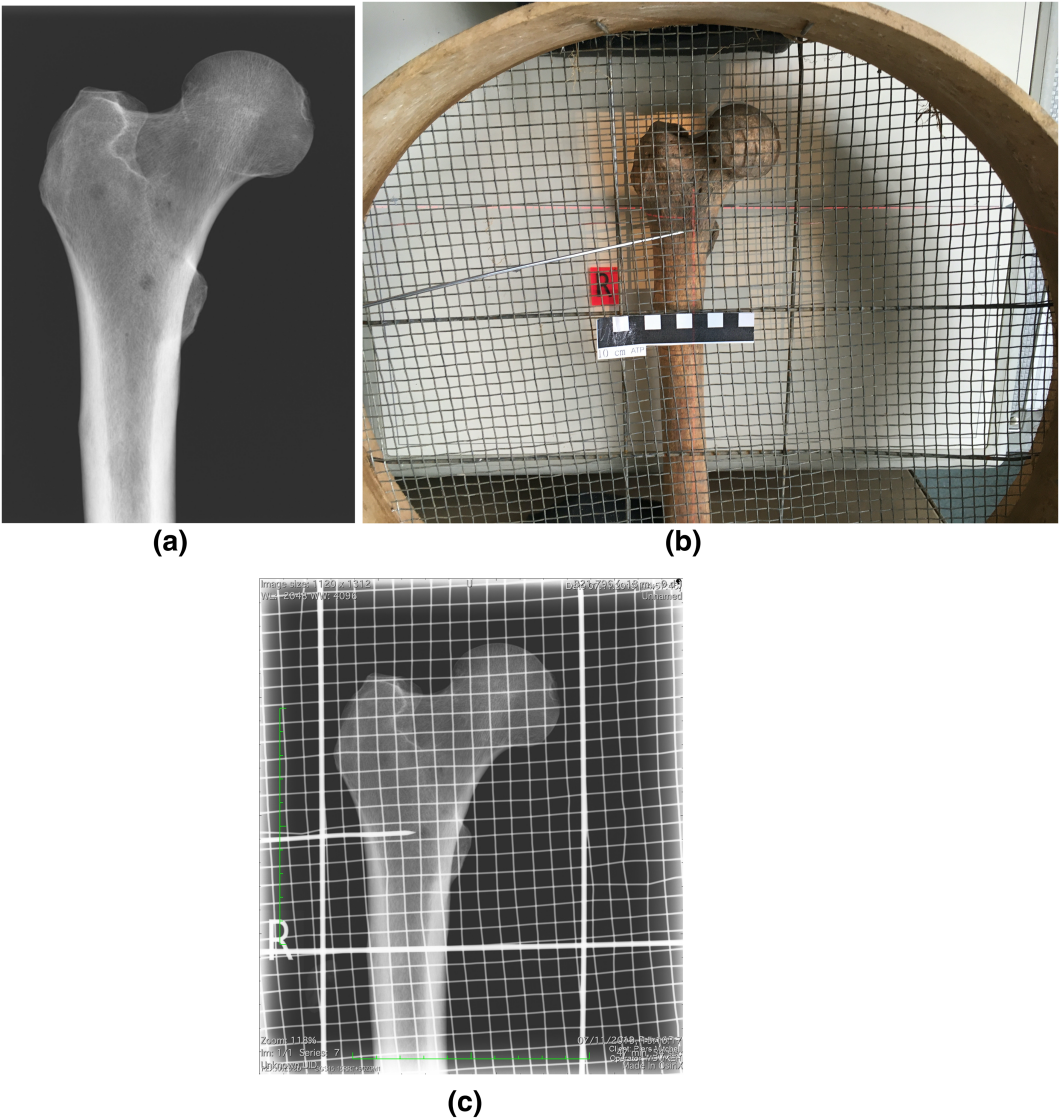
(a) Plain radiograph of right proximal femur of PSN76 showing multiple lytic metastases. (b) Position of the sieve over the femur placed on the digital radiography plate. (c) Radiograph of the femur with pointer highlighting the metastasis prior to marking the bone surface [Colour figure can be viewed at wileyonlinelibrary.com]

An archeological metal mesh designed for coarse sieving (10‐mm mesh size) was laid over the bone and a further radiograph taken. The lesion suitable for biopsy was identified on the radiograph, and the square of the mesh overlying it was determined by counting the squares in both horizontal and vertical axes (Figure 2). A pencil mark was then placed on the surface of the bone overlying the lesion, delineating its margins.

### 2.2 Metal pointer technique

**FIGURE 3:**
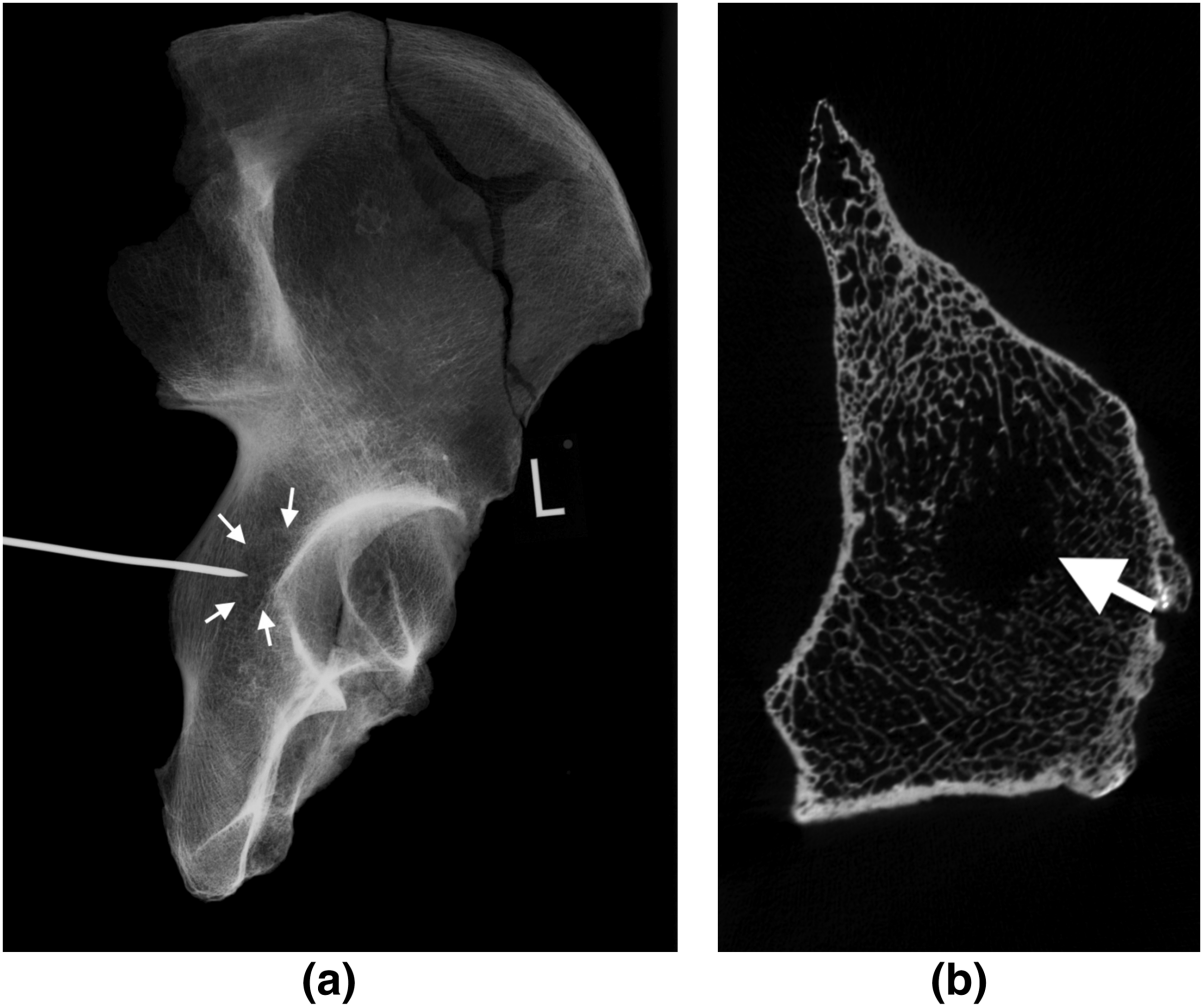
(a) Plain radiograph with the tip of metal pointer located over a lytic metastasis (highlighted with arrows) in left hemipelvis of PSN737, close to the acetabulum; (b) CT shows the metastasis more clearly, located in the cancellous bone (arrow). Image credit: Bram Mulder

**FIGURE 4:**
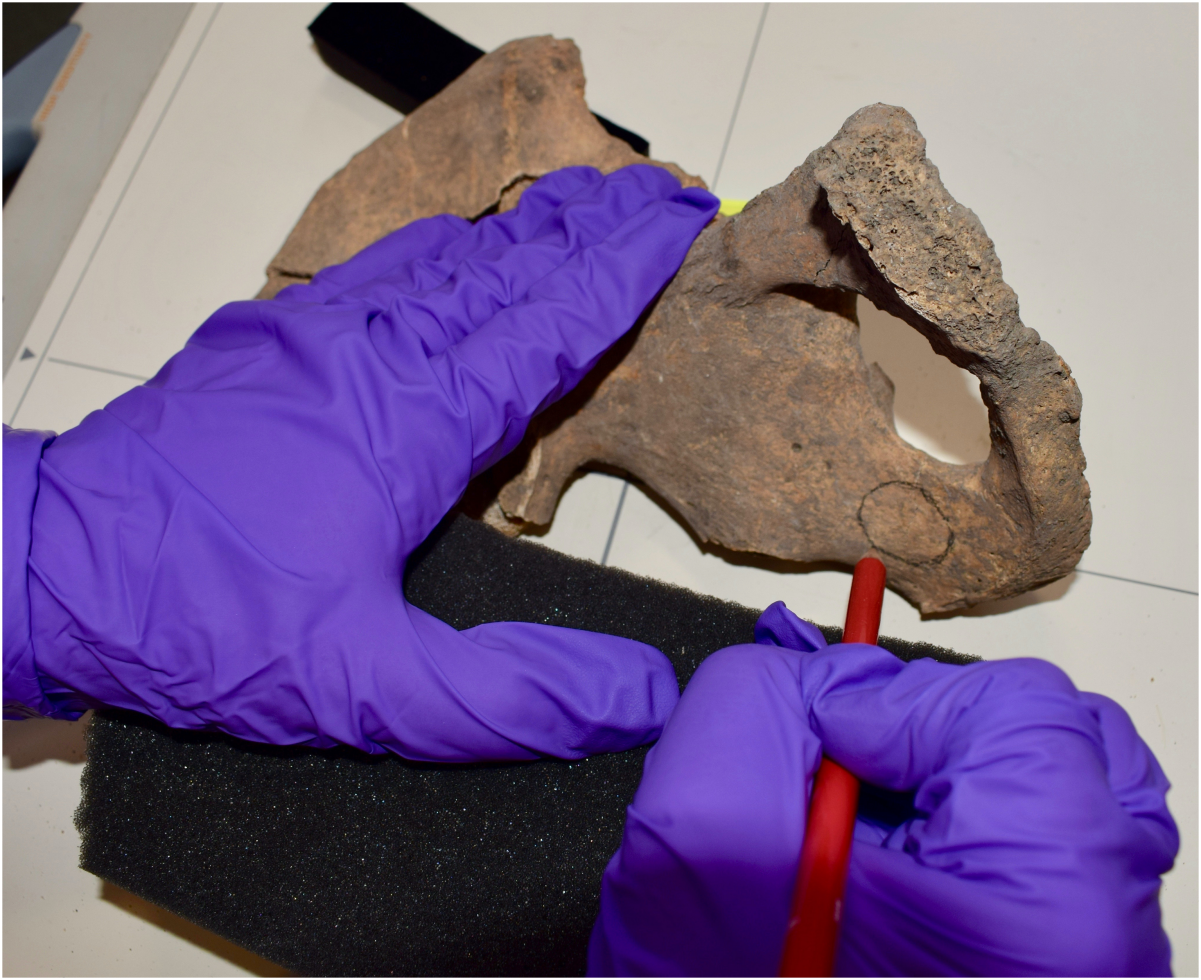
Pencil mark overlying a concealed metastasis within a hemipelvis [Colour figure can be viewed at wileyonlinelibrary.com]

A metal pointer, such as a kebab skewer, was placed on the surface of the bone and a plain radiograph taken. It was then moved progressively closer to the lesion until the tip of the pointer was located at the margin of the lesion (Figure 3). If the lesion was small, then this approach alone was sufficient, but if the lesion was large, this process was repeated four times to delineate all four sides of the lesion (superior, medial, inferior, and lateral borders). The margins of the lesion were then marked with a pencil (Figure 4).

## 3 RESULTS

We found that the grid technique was reliable and required considerably fewer X‐rays to localize the metastases. Often only one or two radiographs were required before the lesion of interest was visualized and identified through the sieve. The pointer technique frequently required at least five radiographs due to repeat positioning of the pointer before the tip was in the required location on all four sides of the lesion. The pointer technique did allow some flexibility in the quantification of larger or irregular shaped metastases, delineating the borders of the metastasis accurately. Therefore, both techniques have merit, but the grid technique requires fewer images to be taken.

## 4 DISCUSSION

Here, we describe two options for how plain radiographs may be helpful in identifying the location of disease lesions present within bone, so that they can be biopsied with accuracy. We compared the ease of use of a grid technique and a pointer technique. We found that both techniques worked well, but the grid technique required fewer images to be taken. This would result in a lower overall radiation dose. If research cost is related to the number of images taken, then the grid technique would also be a more cost‐efficient approach.

We should consider here if the higher number of X‐rays associated with the pointer technique compared with the grid technique might be associated with any drawbacks. Past research in this area suggests that even with cumulative effect, plain radiographs appear unlikely to damage the DNA. A study comparing aDNA yields from archeological human bone that was subjected to zero image, two images, or to 40 repeated images found no significant difference in the recovery and amplification of aDNA (Fehren‐Schmitz et al., 2016). A study comparing microCT and synchrotron X‐ray irradiation showed that radiation doses over 200 Gy did result in reduced aDNA survival, but there was no observable impact when less than 200 Gy was used (Immel et al., 2016).

As for the imaging equipment used in this study, Mark Viner, Director of Reveal Imaging, has confirmed that quality assurance measurements undertaken on the equipment using a Dose Area Product (DAP) meter showed that the maximum exposure used during the study, 55 kV, 2.5 mAs, and a field size of 35 × 43 cm at 1 m, would result in a DAP of 90 mGy/cm^2^.

Based upon this data, it is clear that thousands of radiographs of the same bone would be required to reach a cumulative dose of 200 Gy. As thousands of radiographs would never be required, the available evidence would indicate that we do not need to be concerned about the effects of radiation dose upon the archeological bone using this technique.

### 4.1 Options for biopsy

If a blastic metastasis is to be biopsied, then a portion of the overlying cortex and the center of the lesion can be sampled. However, in a lytic metastasis, there is no remaining tissue in the center of the lesion; therefore, a biopsy should be centered on the margins of the lytic lesion, where cancer cells would have been present infiltrating the cancellous bone prior to death.

Archeological bone is very brittle and friable, so any biopsy method commonly results in bone chunks, crumbled bone fragments, and some bone dust. While this would be a problem if histology is planned, such samples are perfectly suitable for aDNA analysis and proteomics. Sampling should be undertaken following recommended procedures to prevent contamination of the bone sample by the DNA of the researcher taking the sample (Llamas et al., 2017). Instruments that could be used to take the bone biopsy include a circular Dremel wheel of sufficient diameter to cut through the cortex, a rotating hollow core drill 1–2 cm wide (a plug‐cutting drill), an oscillating saw to cut out a square of bone, or a rotating burr to cut through a cortical window to allow the central portion of bone to be levered or scraped out using suitable metal instruments such as an osteotome and curette (Advisory Panel on the Archaeology of Burials in England, 2013). We should note that while these instruments can be used to biopsy bone in the clinical or archeological setting, we did not formally compare the efficacy of each technique on our archeological samples. Therefore, we cannot advise as to which may be most efficient for archeological bones with different cortical thicknesses, such as femora compared with pelvis.

### 4.2 Limitations of the study

This is a proof‐of‐concept paper comparing two options for how to localize a lesion located deep within bone, invisible from surface inspection. Others may have good techniques for undertaking image guided biopsies that differ from those described here. Our aim is to describe the strengths and weaknesses of the two methods we considered and not to claim that other approaches are in any way inferior.

This is not the kind of study that allows statistical comparison of accuracy or reliability of one technique compared with the other. Instead it aims to share practical experience of their ease of use, to determine whether the techniques were effective for the task set, and if there were strengths or weaknesses of either method that came to light from their use.

These techniques require prior training in the interpretation of radiographs to identify pathological lesions. In circumstances where an osteoarcheologist or paleopathologist is not trained in this way, then working with a clinical radiologist from a nearby hospital would be one way to ensure accurate identification of lesions prior to their biopsy.

While lytic cancer metastases that erode the cortical bone will be visible on plain radiographs, those located purely within the cancellous bone will often be a challenge to see or may be completely undetectable using this method (O'Sullivan et al., 2015). In such cases, a better approach would be to use the metal pointer technique while imaging the sample using a microCT scanner, as microCT has been shown to be effective in detecting medullary lesions that may not be visible on plain radiographs (Mitchell et al., 2022).

Biopsy of archeological bone is a destructive technique. While it has become commonplace in certain archeological fields such as isotope analysis and aDNA analysis, the sample will never be the same after the biopsy. All anthropologists need to be aware of the importance of minimizing destruction of ancient samples and just perform biopsy when it is the only technique available to answer a valid research question. Any portion of the biopsy that remains after analysis should be carefully stored for potential future analysis, to avoid the need for repeat biopsy at a later date (Advisory Panel on the Archaeology of Burials in England, 2013).

## 5 CONCLUSION

In this study, we compare the advantages and disadvantages of two techniques that both assist in the localization of lesions that are not visible on the surface of excavated human skeletal remains. We found that both techniques work well, but the grid technique has the advantage of requiring fewer radiographs to be taken. We go on to consider options for taking biopsies of malignant lesions in archeological bone for the purpose of aDNA and proteomic analysis. With this groundwork in place, we look forward to a rapid expansion in research involving the biomolecular analysis of intramedullary lesions in the skeletal remains of past populations.

## CONFLICT OF INTEREST

The authors have no conflicts of interest to declare.
